# Supplementary material for: Are carbon emissions trading and green financial instruments synergistic? -Comprehensive quantitative research based on content analysis
Source: PLoS One. 2024 Mar 7;19(3):e0298601. doi: 10.1371/journal.pone.0298601 (PMC10919601; doi:10.1371/journal.pone.0298601)
Supplement: S1 Data — (ZIP) [file pone.0298601.s001.zip › Data/地级市Z_F工作报告.zip!/║╙─╧/░▓╤⌠╩╨/└┤╫╘╣1⁄2╓┌║┼í╛┬φ┐╦╩2╛▌═°í┐_2007.docx]

各位代表：

　　现在，我代表市人民政府，向大会作政府工作报告，请予审议，并请各位政协委员提出意见。

　　2006年政府工作回顾

　　2006年，在省委、省政府和市委的正确领导下，认真贯彻落实科学发展观，紧紧围绕关注民生、改善民生与构建和谐安阳的要求，深入实施“四路并进”战略，积极顺应宏观调控，大力调整经济结构，着力转变增长方式，较好地完成了市十一届人大三次会议确定的各项目标任务，实现了“十一五”时期的良好开局。

　　——经济实力进一步增强。全市生产总值645.2亿元，增长15.6%，高于全省1.5个百分点。一产增加值97.9亿元，增长7.5%；二产增加值376.9亿元，增长19.3%，其中限额以上工业增加值263.1亿元，增长23.2%，连续4年保持20%以上的高速增长；三产增加值170.5亿元，增长12.4%。人均生产总值达到12054元。

　　——质量效益进一步提升。全市限额以上工业企业实现利润64.1亿元，增长155.6%，高出全省80.6个百分点；综合效益指数217.3%，提高50.1个百分点；财政一般预算收入34.6亿元，增长19.2%。

　　——内外贸易进一步活跃。全社会消费品零售总额167.7亿元，增长15.2%；进出口总值7.7亿美元，增长5.8%。

　　——居民收入进一步提高。城镇居民人均可支配收入10009元，增长13.5%，高于全省1.6个百分点；农民人均纯收入3772元，增长17.1%，高于全省3.5个百分点，连续3年保持两位数增长。

　　一年来，我们主要做了以下工作：

　　(一)立足三农工作，夯实发展基础。坚持用工业的理念谋划农业，综合生产能力显著提高。全市粮食总产295.6万吨，增长16.5%，高于全省6.2个百分点，创历史新高，增产42万吨，占全省增量的近十分之一。一是积极推进农业产业化经营。新建投资3000万元以上的农业龙头企业29家，11家龙头企业进入省重点龙头企业行列，健丰公司“江顺”牌饼干获得中国名牌认证，实现了我市中国名牌产品零的突破。二是着力调整农业结构。建设农业产业化和特色经济乡镇32个，新增高效农业园区70个、高效农业面积16万亩，新建畜禽养殖密集区61个、规模养殖场27个，规模养殖水平居全省前列。三是大力发展劳务经济。积极组织引导农村劳动力外出务工，多渠道增加农民收入，完成农村劳动力转移培训12万人，劳务输出81.3万人，创经济收益42.3亿元。四是不断改善农村生产生活条件。争取上级资金2.2亿元，新建大中型沼气池138座，新增沼气用户5万余户；以改善东部苦水区为重点，较好地解决了10.6万农村人口的安全饮水问题。建设农村超市1000家，为方便农民购物提供了条件。扎实推进马家岩水库、洹河、南海泉治理等工程建设，圆满完成了年度目标。五是积极推进新农村建设。确立了“五年打基础，十年大发展，十五年大突破”的总体目标，开展了“百村示范、千村率先”活动，130个新农村建设试点顺利推进。

　　(二)突出工业强市，加快发展步伐。继续突出工业的主导地位，狠抓工业经济的增长和质量效益的提高。一是大力推进工业振兴工程。支持了50个重点项目和12个工业园区建设，林丰铝业10万吨电解铝、华康大豆蛋白纤维制衣等18个项目相继投产。全市销售收入超亿元的企业达129家、超3亿元的55家、超10亿元的14家。二是着力调整工业结构。全面整合提升钢铁、水泥、纺织等传统行业，大力发展煤化工、电子、装备制造、食品加工等国家鼓励的行业和高新技术产业。安钢350万吨热轧薄板、永兴3500mm中板等项目取得新进展，湖波公司4500吨干法旋窑水泥、昌泰公司5万锭纱锭等项目建成投产；九天化工成功上市，安化20万吨甲醇、鑫龙煤业、凯瑞数码光盘、鑫盛数控机床、宏源型钢车轮总成、永达2万吨鸡肉熟制品等项目顺利实施。三是不断增强自主创新能力。全年科技三项费用支出6727万元，增长31.7%，“大豆纤维功能性研究与开发”和“数控装备中心”被列为省科技型创新资金支持项目，企业技术中心数量达到14家。

　　(三)顺应宏观调控，提高发展质量。认真落实国家宏观调控政策，区别对待、有保有压，有针对性地解决经济运行中出现的矛盾和问题，实现了在调控中的快速发展。一是进一步优化投资结构。按照市场准入政策，对50个不符合条件的新开工项目进行了清理。加大固定资产投资力度，全社会固定资产投资完成310.2亿元，增长39.8%；机械、食品、电子等符合国家产业政策的行业投资得到加强，分别增长91.4%、85.8%和77%；国家重点监测的冶金行业投资得到有效控制，下降13.7%。二是加强资源集约节约利用。强力推进土地“三项整治”和节能减排工作，全市整治土地1908公顷，新增耕地1110公顷，连续8年实现耕地占补平衡；化学需氧量比上年削减1.7%，二氧化硫排放量削减2.7%，单位生产总值能耗下降1.4%。三是大力发展第三产业。打造了殷墟、红旗渠等旅游品牌，全市4A景区达到5个。太行大峡谷景区成功引进战略投资者，实现了我市旅游规模开发的历史性突破。全年接待国内外游客1084.5万人次，旅游收入39亿元，增长10.1%。现代商贸、物流和服务业快速发展，建立了全国最大的免费物流信息网——“八挂来网”，大华、沃尔玛等一批物流项目有序推进。金融业有了新发展，全市金融机构存款余额574.7亿元，增长9.8%，贷款余额332亿元，增长17%，首次实现贷款增幅高于存款增幅，超额完成新增贷款超40亿元争50亿元的目标。四是积极开展银政、银企合作。有效搭建融资平台，先后举办了郑州、安阳银企洽谈会，落实贷款资金77亿元。加强信用担保体系建设，成立了拥有注册资本金6000万元的市信用担保投资有限公司。

　　(四)坚持城乡协调，增强发展后劲。立足当前，着眼长远，不断提高县域和城区经济发展水平。一是积极推进重点项目建设。88项省市重点项目累计投资75.7亿元，完成年计划的103%，一批交通、能源、基础设施和社会事业项目建成或开工，发展后劲进一步增强。安林高速、大广高速安阳段、安楚路、安阳500千伏变电站、安阳至濮阳500千伏输变电等项目建成投运，安南高速、南水北调中线安阳段、河南中国文字博物馆开工建设。二是加强城市规划和基础设施建设。城市总体规划修编顺利通过省级评审，完成了安东新区详细规划和城市水系景观等专业规划。改造治理小巷239条，新增城市绿地100万平方米、新增城市供热66万平方米。市区东、南出口改造工程基本完成，环城河、洪水河治理有序推进，汽车客运南站开工建设。“村村通”工程提前一年完成目标，新改建农村道路1008公里，建成乡镇客运站76个、招呼站429个。创建省级文明城市、国家卫生城市、国家园林城市和全国科技进步先进城市工作进展顺利。三是不断提高城镇化水平。城乡一体化试点工作取得新进展，城镇化率达到34.2%，比上年提高了1.7个百分点，高于全省1.5个百分点，“中州名镇”达到18个，居全省前列。四是大力发展县区经济。五县四区生产总值510亿元，增长18.7%；限额以上工业增加值162.7亿元，增长36%；财政一般预算收入18.9亿元，增长25.3%；占全市的比重分别达到79%、61.8%和54.7%。

　　(五)推动改革开放，激活发展潜力。不断推进重点领域和关键环节的改革，加快发展开放型经济，经济社会发展的活力进一步增强。一是国有企业改革继续深化。全市又有26户企业完成改制，改革面为92%。非公有制经济不断壮大，占全市生产总值的比重达47%。二是其他领域改革全面推进。乡镇机构改革成果得到巩固，农村义务教育管理体制不断完善，县乡财政体制逐步理顺。对104项行政奖励项目进行了清理，精简77项，保留27项。机关事业单位工作人员工资套改顺利实施，企业离退休人员养老金如期调整。农信社、城信社、文化、科技等改革稳妥推进。三是对外开放水平进一步提高。成功举办了第二届世界韩氏恳亲会、殷商文化旅游节、2006跨国企业高层论坛中国药业峰会等大型经贸文化活动，招商引资和对外贸易取得新成果。全市实际利用外资4756万美元，增长67.9%；引进域外资金123亿元，增长99%，分别高于全省17.8和7.4个百分点。

　　(六)注重改善民生，构建和谐社会。坚持以人为本，着力解决人民群众最关心、最直接、最现实的利益问题。圆满完成了向全市人民承诺的十个方面37件实事。一是就业再就业工作得到加强。城镇新增就业人员12.5万人，下岗失业人员再就业6.8万人，“4050”等就业困难人员实现再就业2.5万人，均居全省前列。二是城乡保障体系不断完善。9.4万城市低保对象按新标准施保，10.9万农民享受低保，农村五保集中供养率达20.5%，较好地解决了7500人的温饱问题。三是社会事业全面发展。科技实力不断增强，对经济增长的贡献率达到45%。李官奇、张跃进等发明的4项科技成果获国家科技进步二等奖，占全省所获13项国家科技进步奖的近三分之一。文化事业健康发展，广播电视和新闻出版事业进一步繁荣，殷墟成功列入世界文化遗产保护名录。各级各类教育协调发展，市区3所高中建成招生，农村28.4万名学生享受“两免一补”，资助面达100%。城乡医疗卫生条件明显改善，新型农村合作医疗试点县由1个增加到4个，参合农民259.8万人，参合率达82%。全民健身活动广泛开展，竞技体育水平不断提高，成功举办了第19届中国围棋名人赛及全国中老年太极拳比赛。认真实施公务员法，顺利进行了公务员登记。大力培养和引进人力资源，人才工作得到加强。生态建设成效显著，完成重点工程造林11.6万亩。人口自然增长率5.4‰，继续保持了低生育水平。四是精神文明和民主法制建设取得新进展。社会主义荣辱观教育和精神文明创建活动不断深入，涌现出了王生英、谢延信等先进典型。广泛开展了“转变干部作风，促进工作落实”活动，发展意识、责任意识不断增强。坚持依法治市，自觉接受人大法律监督、工作监督和政协民主监督，认真办理经人大常委会审议通过的11件代表议案和329件人大代表的批评、意见、建议，认真办理政协提案680件。安全生产形势进一步好转，全市各类事故起数和死亡人数分别下降16.4%和20.5%，被评为全省安全生产工作优秀市和煤炭行业先进单位，市消防支队被评为全国公安消防部队英模群体。社会治安防控体系不断完善，信访秩序逐步好转，社会大局保持稳定。双拥共建工作深入开展，军政军民团结进一步加强。切实纠正部门和行业不正之风，认真开展了商业贿赂专项治理，反腐倡廉工作取得新成效。不断加强老区建设，依法保护妇女权益，老龄和关心下一代工作健康发展。人事、民政、统计、审计、史志、档案、经济研究、外事侨务、民族宗教、无线电管理等工作也都取得了新成绩。

　　各位代表！

　　去年是我市在全面建设小康社会、建设豫北区域性中心强市进程中迈出重要步伐、取得显著成绩的一年，也是在土地、资源、环保等约束不断趋紧和区域竞争加剧的情况下，抢抓机遇，开拓进取，保持经济又好又快发展的一年。这些成绩的取得，是市委正确领导的结果，是市人大、市政协大力支持的结果，是全市人民同心同德、团结奋斗的结果。在此，我代表市人民政府，向奋斗在全市各条战线上的广大工人、农民、知识分子、干部、解放军指战员、武警官兵、政法干警以及各界人士，致以崇高的敬意！向所有关心、支持我市现代化建设的港澳台同胞、海外侨胞、国际友人，表示衷心的感谢！

　　在肯定成绩的同时，我们也清醒地认识到，经济社会发展还存在着一些矛盾和问题：工业结构不尽合理，高污染、高能耗、高消耗资源的行业比重较大，高新技术产业、高附加值产品不多；第三产业发展滞后，金融、物流、房地产等新兴服务业发展不快；国企改革的矛盾还比较突出，对外开放程度较低，引进的大企业、大项目不多；多数非公有制企业规模较小，企业核心竞争力不强；县域经济发展不平衡，经济弱县、财政穷县还占有一定的比例；一些政府部门和工作人员，服务意识不强，办事效率不高，经济发展环境仍需要进一步优化；一些干部思想解放不够，小富即安、小进即满，大跨越、高标准发展的意识不强等。对此，我们一定高度重视，在今后的工作中认真加以克服和解决。

　　2007年总体要求和奋斗目标

　　今年是贯彻落实市九次党代会精神的第一年。面对新的发展机遇，我市具备了良好的发展条件。经过多年的积累和发展，拥有了丰富的农副产品资源、独特的文化旅游资源、较好的工业基础和一大批国内外知名企业，地方高速公路快速建设，大广、京珠等国家高速公路穿越境内，具备了快速发展的物质基础。我市南临中原城市群，北接京津冀经济圈，在东部发达地区的先进技术、资金、产业向中西部地区转移中居于有利区位，具备了加快发展的外部条件。市九次党代会提出了“一强五前”、实现“三大转变”、加强“三大建设”的奋斗目标，全市上下团结一心、加快发展的愿望非常迫切，具备了跨越式发展的政治基础。国家实施中部崛起，省加快推进区域协调发展，特别是前不久，省委、省政府在我市召开重要会议，专题研究加快区域经济发展的问题，使我们进一步拉高标杆，找出差距，增强了加快发展的紧迫意识。尽管我市也面临着诸多困难和挑战，但是只要我们进一步解放思想，加压推动，奋力拼搏，只要发挥优势，挖掘潜力，扬长避短，加快发展，就一定能够在区域竞争和新一轮的发展中处于领先位置，进入全省先进行列。

　　今年政府工作的总体要求是：以邓小平理论和“三个代表”重要思想为指导，认真贯彻党的十六届六中全会、省八次党代会和市九次党代会精神，坚持以科学发展观统领经济社会发展全局，紧紧抓住中原崛起和促进区域协调发展重大机遇，深入实施“农业立市、工业强市、三产富市、文化兴市”发展战略，加快推进老工业城市向新型工业强市转变、农产品资源大市向农业产业化强市转变、文化资源大市向文化旅游强市转变，加快推进新型城镇化和“新四创”工作，加快推进改革开放和经济结构调整，注重资源节约和环境保护，注重社会发展和改善民生，突出抓好招商引资、县域经济、非公有制经济发展，为实现“一强五前”奋斗目标奠定坚实基础。

　　今年我市经济社会发展的预期目标是：生产总值增长13%；工业增加值增长17%；地方财政一般预算收入增长14%；全社会固定资产投资增长20%；社会消费品零售总额增长13%；外贸出口增长15%，实际利用外资增长20%；单位生产总值能耗降低5.2%；城镇居民人均可支配收入增长8%，农民人均纯收入增长7%；居民消费价格总水平上涨3%以内；城镇新增就业人数6.5万人，城镇登记失业率控制在4.5%以内；人口自然增长率控制在6.1‰以内。

　　在实现上述目标、争取经济社会跨越式发展的同时，市委、市政府今年还要全力为全市人民办好十个方面44件实事。这十个方面的实事包括农民增收、安全饮水、教育、医疗卫生、城乡就业和社会保障等多个领域，范围更宽，内容也更加充实，既是改善民生的紧迫问题，也是促进经济发展、社会和谐的重要举措。这十个方面的实事已附报告之后，请各位代表一同审议。我们将把这些实事纳入今年的工作目标，加强管理，狠抓落实，真正把这十个方面的实事办成经得起历史和群众检验的民心工程。

　　今年国民经济和社会发展的主要任务

　　完成今年政府工作目标任务，实现经济社会又好又快跨越式发展，必须站在新的历史起点上，坚持高标准，明确新要求，谋求新突破，扎扎实实做好各项工作，努力开创我市经济社会发展新局面。

　　(一)紧紧围绕建设新型工业强市，着力促进工业结构优化升级。坚持以点连线，以线扩面，继续强化工业的主导地位，大力推进工业振兴工程，努力把工业经济提高到一个新水平。一是抓好重点项目建设和重点企业发展。集中用好工业结构调整、高新技术产业化、国债等政府支持、鼓励性资金，加快推进总投资270亿元的96个工业项目建设。确保安化20万吨甲醇和6万吨甲胺、鑫盛1500台精密数控机床、健丰3万吨高档威化饼等37个工业项目建成投产。力争安钢300万吨冷轧薄板、大唐安阳电厂30万千瓦热电联产机组、安锻数控锻压设备、林州重机大型刮板输送机等31个项目尽早开工建设。集中土地、资金、环境容量、重要矿产等资源，全力支持安钢、安彩、安烟、安电、安化等一批重点企业做大做强，尽快培育成为有核心竞争力的大公司、大企业集团。完善全市百户重点企业的发展规划，加大规划的实施力度，力争到2010年全市百户企业销售收入达到2000亿元，占全部工业销售收入的比重达到80%以上。在百户重点企业中，销售收入超百亿元的企业达到2家，超50亿元的10家，超10亿元的40家。二是加快整合提升八大优势行业。在冶金建材行业方面，抓好钢铁、水泥等行业重组整合，调整产品结构，下决心淘汰落后产能和技术，努力建设优质钢、优质铸造生产基地。提高水泥质量和档次，实现规模生产。在装备制造行业方面，加快鑫盛机床、锻压机械、轻工机械等企业的扩建、重组，努力建成国内一流的数控机床、数控锻压设备、大型采煤机械等装备制造业基地。在汽车零部件行业方面，加快汽车零部件企业整合提升，引导零部件企业与汽车制造企业建立战略联盟，力争建成国内重要的汽车底盘零部件生产基地。在煤化工行业方面，加强与永煤集团的合资合作，做好鑫龙煤业集团矿井的改扩建，拉长产业链条，完成以安化、鑫龙煤业和几大焦化企业为主的煤化工生产基地规划。在电子行业方面，支持安彩调整结构，确保液晶玻璃基板中试项目如期开工，支持国内最大的光盘生产企业凯瑞数码快速发展，努力打造国家新型电子显示器件生产基地。在食品及农副产品加工行业方面，依托资源优势，支持健丰饼干、星河植物油等农产品深加工产业快速发展。在纺织和内衣行业方面，力争把大豆蛋白纤维做成全国知名品牌，形成大豆纤维、内衣、巾被、棉纱和面料五个专业纺织产业集群。在医药行业方面，努力盘活现有的800多个药品批号，加快医药企业的重组改造，再现我市医药辉煌。预计到2010年，全市八大行业将实现销售收入1800亿元，占全市规模以上工业的比重由2006年的75%提升到90%。三是扶持壮大县区工业。继续推进柏庄纺织、水冶冶炼、铜冶焦化、姚村汽配、陵阳科技等工业园区建设，壮大凤宝实业、华龙面粉、华康实业、日新钢铁、方快锅炉、豫北金铅等重点企业，培育新的经济增长点和支柱产业，加快县区工业化进程。四是切实加强企业自主创新能力建设。以集成创新、引进创新为主，鼓励企业与高校、科研机构进行技术合作，着力培育一批拥有自主知识产权和发展前景好的高成长性企业，力争液晶玻璃基板、液压模锻锤、宏源型钢车轮总成等重点创新项目的研发取得突破。

　　(二)紧紧围绕农业立市战略，着力抓好农业产业化和新农村建设。坚持把“三农”工作作为重中之重，继续实施“四大兴农计划、三大惠民工程、十大农业产业链、十大增效技术”，大力发展现代农业。一是进一步落实各项支农惠农政策。多方加大“三农”投入，稳定对种粮农民直接补贴，扩大良种补贴、农机具购置补贴规模，加大农业生产资料综合补贴力度，继续实施农村劳动力转移培训、测土配方施肥等补贴政策。二是积极发展现代农业。认真落实农业产业化龙头企业扶持政策，重点培育林州诚泰牧业、汤阴鸿发棉业、滑县种子公司等30家龙头企业，重点支持30个农业产业化示范乡镇和特色农村经济先进乡镇，积极发展50个农产品批发交易市场和农村经济合作组织。深入实施优质粮生产工程，建成国家大型优质小麦生产基地市。围绕粮、棉、油、畜禽、林果等十大农业产业链，发展农产品精深加工，加快农区工业化步伐，配套建设标准化生产基地320万亩，新增高效农业面积20万亩。大力发展现代畜牧业，加快县级动物防疫网络建设，新建标准化养殖密集区30个、规模养殖场15个。三是加快农村基础设施建设。继续推进农村饮水、道路、供电、通讯、沼气等基础设施建设，加快南水北调中线工程安阳段和马家岩水库、洹河河道治理等工程建设进度，开工建设洪水河综合治理工程。改造和建设农村公路400公里，新增农村沼气用户4万户以上，解决8万农村人口安全饮水问题。加大扶贫开发力度，力争完成2.5万人和35个重点村的脱贫任务。四是扎实推进新农村建设。积极探索符合实际的新农村建设模式，在继续抓好生产发展的基础上，重点解决村内道路、给排水设施、垃圾处理等生产生活问题。按照新农村建设标准，力争到2010年全市有1000个村率先实现新农村建设目标。

　　(三)紧紧围绕推进新型城镇化，切实加快城乡基础设施建设。加快中心城区建设步伐，带动卫星城镇和县城建设，全方位、多层次推进城镇化进程。一是深入开展“新四创”工作。以创建省文明城市为龙头，以创建国家卫生城市为基础，以创建国家园林城市为亮点，以创建国家科技进步先进城市为支撑，改革体制，创新机制，重心下移，市区联动，确保“新四创”取得实质进展，进一步提升城市整体文明程度和美誉度。二是加强城市基础设施建设。加大市区路网建设力度，重点实施华祥路立交桥、平原路北段、铁西路改造等新建、改扩建项目，启动中华路北段、铁路以西城区污水管网工程。加快宗村污水处理厂、市医疗垃圾处理厂等建设进度，确保县(市)污水处理工程、垃圾处理工程全面建成投用。高起点规划城区水系，实施美化洹河、洪水河、环城河工程，使安阳尽快成为三面环水、城中有河的宜居城市。加快安南高速、济东高速安阳段建设，实施安新高速拓宽改造工程，积极推动安林高速向山东、山西延伸，尽快与京珠、大广高速形成双十字现代化交通体系。完善城乡公路网，全面推进安姚公路、省道303五陵至岗西段、省道307滑县段等道路建设。三是积极推进城乡一体化进程。在城市基础设施、社会保障体系、户籍管理、环卫、村改居、城乡交通等方面转换体制，统筹城乡和谐发展。加快国家、省、市重点镇建设，进一步整合中心城区和水冶、柏庄、白壁、宝莲寺等卫星镇的经济和社会资源，逐步实现交通、通讯、金融等同城化。积极推进城中村改造，启动县(市)域村镇体系规划编制工作，抓好省级康居示范村建设试点。四是进一步发展壮大县域经济。加强规划和引导，着力发展块状经济。东部以农副产品深加工为重点，实现集中连片开发，拉长产业链条。以汤阴、滑县农畜产品深加工、内黄特色农林产品转化为重点，建设食品加工集聚区。西部以林州陵阳工业园、安阳县工业集中区为依托，建设工业集聚区，争取把水冶、铜冶建成经济社会协调发展、文明富裕的新型工业重镇。认真落实发展壮大县域经济的各项政策，凡是市一级需要下放的权限坚决落实到位，积极为县区招商引资、项目申报搭建平台。大力支持有条件的县(市)跨越式发展，尽快跻身全国百强县和全省经济强县、发展快县行列，鼓励更多的乡镇跻身全省百强乡镇。

　　(四)紧紧围绕建设文化旅游强市，大力发展服务业和社会事业。进一步加大社会事业投入，努力促进经济与社会协调发展。一是大力发展文化事业。充分发挥文化资源优势，积极发展面向基层的公益文化事业，突出抓好城乡基层文化设施建设，建成市图书馆、博物馆，加快建设河南中国文字博物馆，积极筹建市科技馆。大力发展新兴文化产业，培育一批知名文化品牌。加强文化产业项目库建设，搞好文化项目推介和招商工作。大力推进文化体制改革，加快国有文化企业的公司制改造和经营性文化事业单位转企改制步伐，进一步放宽文化市场准入政策，引导和鼓励社会资本兴办文化企业。提高新闻出版、广播影视的质量和水平，加强文化市场和互联网的管理。二是积极发展壮大服务业。抓住殷墟列入世界文化遗产保护名录这一历史机遇，整合文化旅游资源，精心打造黄金旅游线路，积极发展历史文化游、自然风光游、民族精神游，切实把殷墟、红旗渠、周易三个旅游品牌做强做大做优，力争把太行大峡谷打造成5A景区。加快发展交通物流业，在集中力量抓好大华商贸城等重点物流项目的同时，大力发展金融、房地产、信息、咨询、法律、会展等新兴服务业，壮大第三产业的规模和实力，努力实现新的跨越。三是加快发展科技、教育、卫生、体育等社会事业。优化科技资源配置，提高科技成果转化率，加强知识产权保护，加快科技基础设施和科研人才队伍建设。进一步加大对教育的投入，确保各级教育支出的法定增长。重点发展农村教育，全部免除农村义务教育阶段学生的学杂费，继续对困难家庭学生免费提供课本和补助寄宿生生活费。积极发展职业教育，提高高等教育质量，确保安阳师范学院顺利通过国家教育部本科教学评估，加快建设安阳工学院三期工程，做好市体育运动学校、中医药学校整体搬迁和省卫生学校扩建、安阳职业技术学院的筹建工作。健全公共卫生和医疗服务体系，提高疾病预防控制和医疗救治服务能力，大力发展城市社区卫生服务，认真解决好人民群众关心的看病就医问题。严厉打击“医闹”行为，切实维护医护人员的正当权益，创造良好的医疗环境。实施人才强市战略，加快人才使用和开发步伐。深入开展全民健身活动，提高业余训练水平。加强人口和计划生育工作，提高出生人口素质，遏制出生人口性别比升高。做好老龄和关心下一代工作，支持发展慈善和残疾人事业。继续做好人事、民政、统计等工作。

　　(五)紧紧围绕激发内外活力，继续深化改革扩大开放。加大改革攻坚力度，提高对外开放水平，力争在重要领域和关键环节实现新的突破。一是深入推进体制改革。按照因地制宜、分类指导的原则，稳步务实地推进重点领域和关键环节的改革。进一步完善改制政策，基本完成国有工业企业产权制度改革，全面推进商贸、建筑、粮食、交通等非工业企业的改革，尽快实现国有大中型企业主辅分离、辅业改制，稳步推动劣势企业退出市场。加强国有资产监管工作，进一步完善国资监管职能。加快推进城市信用社改制，组建市商业银行。加大发展、利用资本市场工作力度，加快企业上市步伐。继续深入推进以转变政府职能为重点的乡镇机构改革、以落实教育经费保障机制为重点的农村义务教育改革、以增强基层财政保障能力为重点的县乡财政管理体制改革、以建立新型金融组织为重点的农村信用社改革等一系列农村综合改革。认真组织实施收入分配制度改革，进一步规范机关事业单位人员津贴补贴标准。继续推进科技、教育、卫生、体育等其它领域的改革。二是加快发展开放型经济。加强与美国贝克菲尔德市、日本草加市、加拿大莱桥市等国外友好城市的交往与交流，争取在对外交往和利用外资上取得新进展。充分发挥我市环渤海、中原经济协作区成员市的优势，加强与邯郸、聊城、长治等周边城市的交往，在共同开发资源、产业协作配套上开展深度合作。全力抓好招商引资，高度重视大项目和战略投资者的引进。充分利用市政府驻长三角、珠三角、环渤海及中原地区4个经济发展联络处的作用，积极开展以商招商、小分队招商和针对性招商等多形式的对外招商活动，特别是瞄准世界500强和国内著名企业，在引进大项目上下功夫。积极参加第二届中国中部投资贸易博览会、厦洽会、乌洽会、高交会等大型商贸活动，加强签约项目的跟踪落实，多形式、多渠道引进域外资金、项目、技术和人才。三是加快发展非公有制经济。充分调动全社会的发展积极性，进一步挖掘各个方面的投资潜力，放心、放胆、放手发展非公有制经济。认真落实促进非公有制经济发展的各项政策措施，重点抓好100家重点非公有制企业的发展和规模提升，加快形成企业集群，组建企业集团，力争今年新增销售收入10亿元以上的非公有制企业达6家以上。

　　(六)紧紧围绕建设资源节约型和环境友好型社会，扎实推进节能降耗和环境保护工作。把节约资源和保护环境作为推进经济转型的重要抓手，强化目标责任，确保完成节能降耗和污染减排任务。一是强力推进节能降耗。建立健全节能降耗目标责任制，通过综合运用产业政策、环境容量、差别电价和严格准入门槛等措施，着力抓好冶金、电力、化工、建材等高耗能行业和重点耗能企业的节能降耗。大力发展循环经济，加快推进建筑、交通、运输、商业等领域节能技术、节能产品的研发和应用。二是强化土地集约节约利用。严格控制新增建设用地规模，严格执行新的土地有偿使用费标准，严格实行土地出让“招拍挂”制度。以工业集聚区为重点，大力推广使用多层标准厂房，减少企业非生产性用地，加快推进土地“三项整治”，提高单位土地面积投资强度。三是加强环境保护和生态建设。全面落实环境影响评价、污染物排放总量控制、环境目标责任制等一系列环境保护制度，推进重点流域区域环境治理，严格控制新污染源，有效削减主要污染物排放总量。化学需氧量比上年下降3.5%，二氧化硫排放量削减9.2%。继续实施退耕还林、太行山绿化、防沙治沙等重点工程，进一步改善生态环境。

　　(七)紧紧围绕构建和谐安阳，认真解决关系人民群众利益的问题。坚持以人为本，改善民生，强力推进人民群众最关心、最直接、最现实利益问题的解决。一是认真做好扩大就业工作。加强职业技能培训，建立经济增长与扩大就业协调推进机制，创造更多的就业岗位，加大对下岗失业人员、“4050”人员、城镇低保人员、零就业家庭等困难群体再就业的帮扶力度。继续做好高校毕业生、进城务工农民和被征地农民的就业工作。二是不断完善社会保障体系。进一步完善城镇职工基本养老、失业、医疗、工伤等保险制度，建立健全统筹层次高、保障实力强、覆盖范围广的城乡社会保障体系，参加基本养老保险新增1.7万人、基本医疗保险新增2.5万人。切实关心弱势群体，做好城乡居民最低生活保障工作，加大以扶老、助残、救孤、济困为重点的社会救助力度。全面普及新型农村合作医疗，在4个县的基础上，今年覆盖到内黄县及4个城区的所有农村人口。三是高度重视安全生产工作。全面落实安全生产责任制，加大对违法、非法生产的打击力度，切实抓好煤矿、非煤矿山、危险化学品、道路交通、烟花爆竹及消防安全等重点领域的专项整治，严防重特大事故的发生。加强食品药品监管，确保人民群众饮食和用药安全。四是切实做好社会稳定工作。健全社会利益协调机制，加强和改进信访工作，畅通群众诉求渠道，引导群众通过理性合法形式表达利益要求。大力推进平安安阳建设，加强社会治安综合治理，加快城市报警与监控系统建设步伐，构建灵活多样的群防群治网络，严格依法打击各类犯罪活动，维护社会安全和稳定。

　　(八)紧紧围绕提高全民素质，大力加强社会主义精神文明和民主法制建设。深入开展理想信念和传统道德教育，大力弘扬自力更生、艰苦奋斗的红旗渠精神。加强社会公德、职业道德、家庭美德和诚信教育，推进文明城市、文明村镇、文明社区和文明行业创建活动。加强未成年人和大学生思想道德建设，努力构建学校、社会、家庭“三位一体”的未成年人思想道德教育网络。坚持依法治市，提高全民法制素质。严格执行人大及其常委会的决议、决定，认真抓好《监督法》的贯彻学习，自觉接受人大及其常委会的工作监督和法律监督，支持人大代表依法行使职权，接受人民政协的民主监督。密切与各民主党派、工商联、无党派人士的联系，发挥工会、共青团、妇联等人民团体的桥梁纽带作用。加强国防动员、人民防空和后备力量建设，重视国防设施保护，支持驻安部队建设，深入开展双拥共建活动，巩固和加强军政军民团结，力争“全国双拥模范城”创建成功。

　　加强政府自身建设

　　新时期经济社会的发展，对政府改革和建设提出了新的更高的要求。我们必须进一步加强自身建设，创新体制机制，提高工作效率和服务水平，增强政府执行力和公信力，建设人民满意的政府。

　　(一)坚持执政为民，建设服务型政府。继续深化行政管理体制改革，加快职能转变，积极为各类市场主体提供优质服务，创造良好发展环境。在搞好经济调节和市场监管的同时，切实加强社会管理和公共服务职能。继续办好“12345”市长热线和效能监督热线，及时了解民意民声，帮助群众解决各种实际困难。加强应急管理体系建设，有效应对自然灾害、事故灾难、公共卫生和社会安全事件，提高应急管理和抗风险能力。大力推进政务公开，确保权力透明运行。全面推行学校、医院、供水、供电等公用事业单位办事公开制度，保障人民群众的知情权、参与权和监督权。加快电子政务建设，完善政府门户网站。进一步规范和减少行政审批，简化办事程序，完善限时办结、首问负责、服务承诺等制度，提高办事效率，更好地为基层、企业和公众服务。

　　(二)严格依法行政，建设法治型政府。认真贯彻《行政许可法》和《全面推进依法行政实施纲要》，更新行政理念，进一步提高依法行政水平。建立健全符合科学发展观要求的经济社会发展综合评价体系和考核指标体系，开展政府绩效评估，建立行政问责制度。加强政府法制工作，强化行政执法监督，推进行政执法责任制，搞好行政复议。规范行使行政权力，加强审计监督、效能监察，做到有权必有责、用权受监督、违法要追究。在土地征用、房屋拆迁、企业改制等方面，严格按法定程序办事，切实纠正损害群众利益的行为。健全长效机制，落实各项责任制，坚决查处“四乱”和“吃拿卡要”行为，下功夫治理和优化经济发展环境。抓好政府工作人员的教育、监督和廉洁自律，加强对重点领域的监管，严肃查处各类违纪违法案件，创新反腐倡廉工作体制机制，深入开展党风廉政建设和反腐败斗争。

　　(三)改进工作作风，建设务实型政府。深入开展“讲正气、树新风”主题教育活动，进一步增强忧患意识、公仆意识、节俭意识，全面加强五个方面的作风建设，树立八个方面的良好风气，切实把各级干部的精力引导到抓发展上来。弘扬求真务实精神，大兴求真务实之风，真正做到亲民、务实、进取、廉洁。增强工作责任感和紧迫感，始终保持只争朝夕、奋发有为的精神状态，集中时间和精力，多做一些事关全局、谋划长远、夯实基础的工作。积极开展调查研究，切实解决影响改革发展稳定的突出问题，主动为人民群众排忧解难。牢记“两个务必”，坚决抵制拜金主义、享乐主义和奢靡之风。坚持勤俭节约，严格财政支出管理，严格公费出国出境管理，严格规范公务接待行为，真正把有限的资金和资源用在刀刃上。大力精简会议、文件，切实改进会风、文风，进一步清理评比达标表彰活动。树立终身学习思想，不断更新知识，提高理论素养和业务能力，建设学习型机关。进一步提高公务员管理的法制化、规范化水平，努力建设一支政治坚定、业务精通、清正廉洁、作风优良的公务员队伍。

　　各位代表！

　　风劲潮涌，自当扬帆破浪；任重道远，更需快马加鞭。新的形势催使我们加速发展，新的任务亟需我们真抓实干。让我们在以胡锦涛同志为总书记的党中央领导下，高举邓小平理论和“三个代表”重要思想伟大旗帜，团结奋进，求真务实，开拓创新，勤廉为民，苦干争先，为圆满完成今年的各项目标任务，为全面建设富裕、文明、和谐的新安阳而努力奋斗！
